# 

**Published:** 1861-01

**Authors:** 


### LIST OF COLLABORATORS.

ARMOR, S. G., M.D.,
Late Professor of Pathology and Clinical Medicine, Missouri Medical College.

ATKINSON, WM. B., M.D.,
Lecturer on Obstetrics, and Diseases of Women and Children, Philadelphia.

ATLEE, JOHN L., M.D.,
Late President Pennsylvania State Medical Society.

ATLEE, WALTER F., M.D.,
Philadelphia.

BARCLAY, ROBERT G., M.D.,
Jerusalem, Syria.

BELL, JOHN, M D.,
Philadelphia, formerly Professor of Medicine, Medical College of Ohio.

BEMISS, S. M., M.D.,
Professor of Clinical Medicine, University of Louisville.

BLACKBURN, L. P., M.D.,
Natchez, Miss.

BLACKIE, GEORGE C., M.D.,
Professor of Botany, University of Nashville.

BLACKMAN, G. C., M.D.,
Professor of Surgery, Medical College of Ohio.

BOBBS, J. L., M.D.,
Formerly Professor of Anatomy, Indianapolis.

BOWEN, W. S., M.D.,
New York.

BOZEMAN, N., M.D.,
Montgomery, Ala.

BRADFORD, J. T., M.D.,
Augusta, Ky.

BRINTON, JOHN H., M.D.,
Lecturer on Operative Surgery, Philadelphia.

BUMSTEAD, FREEMAN J., M.D.,
New York.

CARNOCHAN, J. M., M.D.,
Professor of Surgery in the New York Medical College.

CHIPLEY, W. S., M.D.,
Professor of Medicine, Transylvania University.

CLAPP, A., M.D.,
New Albany, Indiana.

CLIFFE, D. B., M.D.,
Franklin, Tenn.

DA COSTA, J., M.D.,
Lecturer on the Practice of Medicine at the Philadelphia Medical Association.

DICKSON, SAMUEL H., M.D.,
Prof. of Medicine, Jefferson Medical College.

DUNGLISON, RICHARD J., M.D.,
Physician to the Burd Orphan Asylum, and to the Albion Society, Philadelphia.

DUPIERRIS, M., M.D.,
Havana, Cuba.

EDGAR, W. F., M.D.,
United States Army.

FARRAR, S. C., M.D.,
Jackson, Miss.

FENNER, C. S., M.D.,
Memphis, Tenn.

FISCHER, EMIL, M.D.,
Philadelphia.

FLINT, AUSTIN, M.D.,
Professor of Clinical Medicine, Auscultation, and Percussion, New Orleans School of Medicine.

GADBURY, W. Y., M.D.,
Benton, Miss.

GIBBS, O. C., M.D.,

Chautauque County, New York.

HALL, J. P., M.D.,
Glasgow, Ky.

HAMMOND, WM. A., M.D., U.S.A.,
Prof. of Anat. in the University of Maryland.

HARDIN, JOHN, M.D.,
Professor of Obstetrics, Kentucky School of Medicine, Louisville.

HARTSHORNE, EDWARD, M.D.,
Surgeon to the Pennsylvania Hospital.

HASKINS, E. B., M.D.,
Professor of Medicine, Shelby Medical College, Nashville, Tenn.

HENTZ, C. A., M.D.,
Marianna, Florida.

HEWSON, ADDINELL, M.D.,
One of the Surgeons to Wills Hospital for Diseases of the Eye, Philadelphia.

HOLLINGSWORTH, SAMUEL L., M.D.,
Formerly Editor of the Medical Examiner, Philadelphia.

JEWELL, WILSON, M.D.,
Philadelphia, formerly President of the Quarantine and Sanitary Convention.

KEATING, WM. V., M.D.,
Lecturer on Obstetrics and the Diseases of Women, at the Phila. Medical Association.

KERR, JOHN G., M.D.,
Canton, China.

KUNKLER, G. A., M.D.,
Madison, Ia.

LOGAN, BEN., M.D.,
Shreveport, La.

LOPEZ, A., M.D.,
Mobile, Ala., formerly President of the Alabama State Medical Society.

MEIGS, J. AITKEN, M.D.,
Professor of the Institutes of Medicine in the Medical Department of Penna. College.

MITCHELL, S. WEIR, M.D.,
Lecturer on Physiology at the Philadelphia Medical Association.

MOREHOUSE, GEORGE R., M.D.,
Philadelphia.

PACKARD, JOHN H., M.D.,
Philadelphia.

RAPHAEL, B. I., M.D.,
Professor of the Principles and Practice of Surgery and Surgical Pathology, in the New York Medical College and Charity Hospital.

REEVE, J. C., M.D.,
Dayton, Ohio.

REEVES, JAMES E., M.D.,
Fairmount, Virginia.

RIVES, L. C., M.D.,
Formerly Professor of Midwifery, Medical College of Ohio.

SMITH, J. LAWRENCE, M.D.,
Professor of Chemistry, University of Louisville.

SNEED, W. C., M.D.,
Frankfort, Ky., formerly President of Kentucky State Medical Society.

SPILMAN, C. H., M.D.,
Harrodsburg, Ky., formerly President of Kentucky State Medical Society.

STORER, HORATIO R., M.D.,
Formerly one of the Physicians to the Boston Lying-in Hospital, Boston, Mass.

SUTTON, GEO., M.D.,
Aurora, Ia.

SUTTON, W. L., M.D.,
Georgetown, Ky., formerly President Kentucky State Medical Society.

THOMPSON, S., M.D.,
Albion, Ill., formerly President of the Illinois State Medical Society.

TODD, L. BEECHER, M.D.,
Lexington, Kentucky.

WILLIAMS, E., M.D.,
Cincinnati, Ohio.

### Bi-Monthly Bibliographical Record.

AMERICAN.

Lives of Eminent American Physicians and Surgeons of the Nineteenth Century. Edited by S. D. Gross, M.D., etc. 8vo, pp. 836. Philadelphia: Lindsay & Blakiston, 1860. (From the publishers.)

Statistical Report of the Sickness and Mortality of the Army of the United States, from January, 1855, to January, 1860. Prepared by R. H. Coolidge, M.D. 4to., pp. 515. Washington, 1860. (From the Surgeon-General.)

On Diseases Peculiar to Women, including Displacements of the Uterus. By Hugh L. Hodge, M.D., etc. 8vo., pp. 469. Philadelphia: Blanchard & Lea, 1860. (From the publishers.)

The True Physician; an Anniversary Discourse delivered before the New York Academy of Medicine. By John Watson, M.D. 8vo., pp. 44. New York: S. S. & W. Wood, 1860. (From the author.)

A Practical Treatise on Mechanical Dentistry. By J. Richardson, M.D., D.D.S., etc. 8vo., pp. 427. Philadelphia: Lindsay & Blakiston, 1860. (From the publishers.)

Transactions of the Fifteenth Annual Meeting of the Ohio State Medical Society. 8vo., pp. 267. Columbus, 1860. (From the Secretary.)

Proceedings of the American Pharmaceutical Association, at the Ninth Annual Meeting, held in New York, Sept. 1860. 8vo., pp. 278. Philadelphia, 1860. (From the Secretary.)

Quackery Unmasked; or a Consideration of the Most Prominent Empirical Schemes of the Present Time. By Dan. King, M.D., etc. 12mo., pp. 334. New York: S. S. & W. Wood, 1860. (From the publishers.)

The Physician's Diary for 1861. Buffalo: Breed, Butler & Co., 1861. (From the publishers.)

Report on Insanity. By Richard Gundry, M.D., etc. Pamphlet, 8vo., pp. 59. Columbus, 1860. (From the author.)

Introductory Discourse on Speculative and Inductive Medicine. By Henry Hartshorne, M.D., etc. 8vo., pp. 19. Philadelphia, 1860. (From the author.)

Introductory Discourse on the Classification of the Animal Kingdom. By D. Hayes Agnew, M.D., etc. 8vo., pp. 16. Philadelphia, 1860. (From the author.)

An Address delivered before the American Medical Association, at the Thirteenth Annual Meeting. By Henry Miller, M.D., etc. Reprint, 8vo., pp. 28. Philadelphia, 1860. (From the author.)

Introductory Lecture to the Course on the Institutes of Medicine, in Jefferson Medical College. By Robley Dunglison, M.D., etc. 8vo., pp. 30. Philadelphia, 1860. (From the author.)

Proceedings of the Academy of Natural Sciences of Philadelphia. 8vo., pp. 361–516. Philadelphia, 1860. (From the Secretary.)

First Report to the Cotton Planters' Convention of Georgia on the Agricultural Resources of Georgia. By Joseph Jones, M.D., etc. 8vo., pp. 312. 1860. (From the author.)

An Inquiry concerning the Value of Testimony respecting Facts as they appear to a Mind partly Conscious. By T. L. Wright, M.D. Reprint, 8vo., pp. 32. Columbus, 1860. (From the author.)

Observations upon the Form of the Occiput in the various Races of Men. By J. Aitken Meigs, M.D., etc. Reprint, 8vo., pp. 18. Philadelphia, 1860. (From the author.)

An Introductory Lecture, delivered in the University of Maryland. By Edward Warren, M.D., etc. 8vo. Baltimore, 1860. (From the author.)

FOREIGN.

Compendium of Human Histology. By C. Morel, M.D., etc. Translated and edited by W. H. Van Buren, M.D., etc. 8vo., pp. 207. New York: Baillière Brothers, 1861. (From the publishers.)

On the Reparative Process in Human Tendons after Subcutaneous Division for the Cure of Deformities. By William Adams, F.R.C.S., etc. 8vo., pp. 175. London: Churchill, 1860. (From the author.)

Chemistry in its Relations to Physiology and Medicine. By G. E. Day, M.D., etc. 8vo., pp. 524. London: Baillière, 1860. (From the publisher.)

Cellular Pathology as Based upon Physiological and Pathological Histology. By Rudolf Virchow. Translated by Frank Chauce, M.B., etc. 8vo., pp. 511. London: Churchill, 1860.

Maximen der Kriegsheilkunst. Von Dr. L. Stromeyer 8vo., pp. 150. Hanover, 1861.

Traité des Maladies des Voies Urinaires. Par C. Phillips, M.D., etc. 8vo., pp. 676. Paris: Germer Baillière, 186 .

The Pocket Anatomist: being a Complete Description of the Anatomy of the Human Body, for the Use of Students. By M. W. Hilles. 12mo., pp. 263. Philadelphia: Lindsay & Blakiston, 1860. (From the publishers.)

Della Odierna Diminuzione della Podagra e delle sue Cause Saggio di Patologia Storica. Del A. Corradi, M.D., etc. 4to., pp. 58. Bologna: Gamberini, 1860.

Advice to a Mother on the Management of her Offspring. By P. H. Chavasse, F.R.C.S. Fifth edition. Fcap 8vo., pp. 255. London: Churchill, 1860.

